# Enzymatic Modification of Walnut Shell for High-Efficiency Adsorptive Methylene Blue Removal

**DOI:** 10.3390/ma18153434

**Published:** 2025-07-22

**Authors:** Xifeng Lv, Xuejian Zhou, Ruiqi Yang, Di Cai, Wenqiang Ren

**Affiliations:** 1 Key Laboratory of Modern Agricultural Engineering, College of Chemistry and Chemical Engineering, Tarim University, Alar 843300, China; lvning7431@163.com (X.L.); 15511569770@163.com (X.Z.); 17615349985@163.com (R.Y.); 2 National Energy R&D Center for Biorefinery, Beijing University of Chemical Technology, Beijing 100029, China; 3 Research Center for Eco-Environmental Sciences, Chinese Academy of Sciences, Beijing 100085, China

**Keywords:** walnut shell, peroxidase, hydrothermal carbonization, methylene blue, adsorption

## Abstract

Developing energy-efficient and environmentally benign synthesis protocols is crucial to agricultural waste-based adsorbent preparation. This study prepared novel walnut shell-derived adsorbents by enzymatic modification using a green process, and the as-prepared material was used for methylene blue (MB) removal from wastewater. The results showed that under the optimized conditions (100 mg L^−1^ methylene blue (MB) solution, pH 7, 30 °C, 120 min adsorption time, and 0.14 g adsorbent dosage), WS-1 exhibited an MB removal efficiency of 93.67%, which was only slightly lower than that of WS-2 that was prepared by further carbonization of WS-1 using the low-temperature hydrothermal method (99.01%). Kinetic analysis confirmed WS-1 exhibited pseudo-second-order adsorption kinetics, which were generally similar to those of WS-2. However, the results obtained by the isotherm model followed by the Langmuir model of WS-1 indicated monolayer adsorption involving combined weak chemisorption and physisorption, which was different from the WS-2 (followed the Freundlich model that inferred multilayer chemisorption). In conclusion, this study successfully converted walnut shells, a type of agricultural waste, into functional adsorbents by a novel, simple, and greener enzymatic modification method, thereby achieving dual benefits of waste valorization and wastewater treatment.

## 1. Introduction

Methylene blue (MB), a prevalent industrial dye, is commonly treated through biological degradation [1], membrane separation [2], ultrafiltration [3], adsorption [4], and solvent extraction [5]. Among the methods, adsorption has gained prominence for dye removal due to its operational simplicity, high efficiency, and cost-effectiveness [6]. The key to achieve highly effective MB removal from wastewater by adsorption is to develop a low price and facile regeneration adsorbent [7]. On this basis, various types of adsorbent materials have been suggested for the specific adsorption of MB.

Among the candidates, lignocellulose-derived materials, especially biochar, have emerged as a preferred adsorbent due to their environmental compatibility, low production cost, and straightforward regeneration [8]. However, the pristine biochar obtained by typical carbonization generally exhibits limited adsorption capacity of organic dyes compared to modified dyes, owing to the low specific surface area and low amount of reactive surface functional groups, necessitating structural modifications for performance enhancement [9,10].

For the modification of pristine biochar to improve MB adsorption efficiency, various studies have been performed in past years. For instance, Fang et al. [11] developed humic acid-sucrose-modified red mud (RM-SC-HA) with an intrinsic adsorption capacity of 417.12 mg/g for methylene blue (Langmuir model, 298 K), and an experimentally validated value of 404 mg/g for a 100 mg/L solution of methyl blue. The removal of methyl blue from the simulated industrial wastewater exceeded 90%, and it demonstrated magnetic recovery and regeneration. Ren et al. [12] pioneered systematic modification of Sphagnum moss using HNO_3_-H_2_SO_4_ treatment, preparing a stable biosorbent with 116.9 mg/g adsorption capacity, representing >30% improvement over the unmodified material. In another study, Wei et al. [13] employed H_2_O_2_ modification to enhance wood-derived hydrochar (HTC), achieving an 11.20% increase in surface area and 11.80% increase in cation exchange capacity. The HTC-H variant demonstrated enriched oxygen-containing functionalities (hydroxyl, carboxyl groups) and 45.70% higher MB adsorption capacity (33.19 mg/g) compared to the pristine HTC. While modified adsorbents demonstrate efficacy in dye removal, the industrial-scale implementation of candidate adsorbents remains constrained by a series of significant barriers, such as the elevated feedstock costs, energy-intensive processing, and secondary contamination risks.

To address these challenges, we developed a sustainable preparation strategy using walnut shells (WSs), a type of low-price agricultural waste, as the template of adsorbents [14]. WSs exhibit advantageous characteristics for modification, including high carbon content, well-developed pore structures, abundant surface functional groups, and cost-effective availability [15], which allow for effective modifications even under relatively warm conditions. For instance, enzymatic catalysis provides an eco-efficient approach for biomass modification. Lignin peroxidase, for example, specifically cleaves β-O-4 linkages in lignin–carbohydrate complexes under mild conditions and could selectively delignify the biomass matrix to enhance surface accessibility [16].

**Figure 1:**
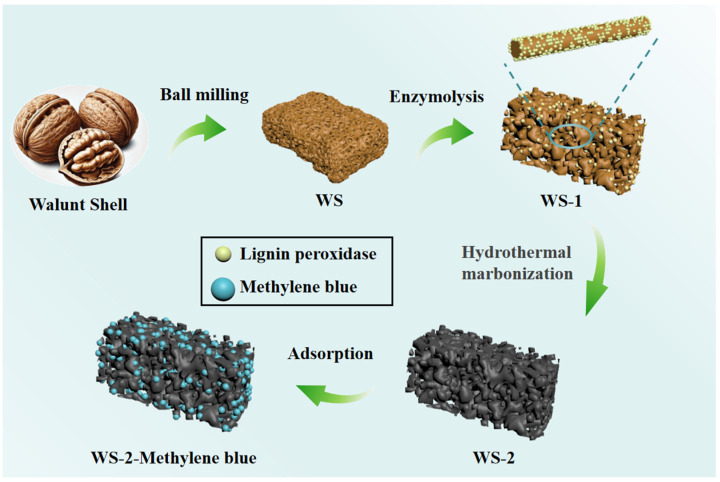
Schematic illustration of the synthesis protocol for WS-1 and WS-2.

As shown in Figure 1, in this study, a novel biobased adsorbent was prepared by enzymatic modification of WSs under greener and milder conditions (code WS-1). The performances of the adsorbent in MB adsorption were comprehensively investigated. To obtain better performances, we further carbonized the WS-1 by hydrothermal processes (code WS-2), and the kinetics of the two candidate adsorbents were analyzed. The good performances of MB adsorption using the WS-derived materials suggested the novel green protocol for adsorbents could contribute to a cost-effective dye wastewater treatment, and showed promise for realistic applications.

## 2. Materials and Methods

### 2.1. Materials

Citric acid (CA) was procured from Tianjin Zhiyuan Chemical Reagent Co., Ltd. (Tianjin, China), MB was procured from Tianjin Beichen Fangzheng Reagent Factory (Tianjin, China), 95% ethanol was obtained from Tianjin Bohua Chemical Reagent Co., Ltd. (Tianjin, China), and lignin peroxidase was purchased from Tianjin Bohua Chemical Reagent Co., Ltd. (Tianjin, China). WS was sourced from local markets in Alar City, Aksu Prefecture, Xinjiang Uygur Autonomous Region.

### 2.2. Preparation of Adsorbents

For the preparation of adsorbents, the WSs underwent sequential pretreatment, including (1) repeated deionized water washing to remove surface impurities, (2) oven-drying at 105 °C until constant mass was reached, (3) mechanical grinding using a planetary mill, and (4) sieving to obtain 50-mesh particles. In the enzyme modification process, 25 g of pretreated walnut shells (WSs) was mixed with 5% (*w*/*w*) lignin peroxidase (LiP) in 150 mL of deionized water. The mixed suspension was incubated in a thermostatic magnetic stirrer (40 °C, 150 rpm) for 48 h.

To maintain the catalytic activity of LiP, the suspension was continuously supplemented with 0.5 mM H_2_O_2_ every 12 h. During this process, LiP was immobilized on the surface of WS particles mainly through physical adsorption. After termination of the reaction, the product was washed thoroughly with distilled water until a neutral pH was reached to remove the unabsorbed free enzyme, followed by drying in an oven at 70 °C for 3 h to obtain WS-1. Hydrothermal carbonization was carried out under a N_2_ atmosphere. In this process, a solution of WS-1 with 2 M citric acid (solid–liquid ratio 1:10) was added to a PTFE-lined autoclave, which was sealed and then vented with N_2_ for 5 min. Temperature was maintained at 180 °C for 5 h. At the end of the reaction, the product was sequentially washed twice using 95% ethanol and distilled water until a neutral pH was reached, and finally dried at 70 °C for 3 h to obtain hydrothermal-carbonized walnut shells (WS-2).

### 2.3. Characterization

MB concentration was quantified using a UV-2800 double-beam spectrophotometer (Shanghai Sunny Hengping Scientific Instrument Co., Ltd., Shanghai, China) with calibration in the 550–700 nm wavelength range. Morphological characteristics were analyzed by field-emission scanning electron microscopy (FE-SEM, JSM-7800F, JEOL Ltd., Tokyo, Japan) at 5 kV accelerating voltage with gold sputter coating. Surface area, pore volume, and pore size distribution were determined via nitrogen physisorption at 77 K using an Autosorb-iQ analyzer (Quantachrome Instruments, Boynton Beach, FL, USA, model 4000e). The pore size distribution was calculated from the adsorption branch of the isotherm using the Barrett–Joyner–Halenda (BJH) model. Functional group analysis was performed on an IRTracer-100 Fourier Transform Infrared spectrometer (Shimadzu Corp., Kyoto, Japan) using the KBr pellet method (4 cm^−1^ resolution, 32 scans) across 400–4000 cm^−1^. Crystalline phase identification was conducted via X-ray diffraction (X-ray Diffraction, D8 Advance, Bruker AXS, Karlsruhe, Germany) with Cu Kα radiation (λ = 1.5406 Å, 40 kV, 40 mA) over 5–80° 2θ range. Thermal stability was evaluated by thermogravimetric analysis (TGA 209 F3 Tarsus, NETZSCH, Selb, Germany) under nitrogen atmosphere (20 mL min^−1^) with 10 °C min^−1^ heating rate from 30 to 800 °C.

### 2.4. Adsorption Experiments

The adsorption performance was systematically evaluated against critical operational parameters including pH, temperature, contact time, and adsorbent dosage. Batch experiments were performed using 150 mL conical flasks containing 50 mL of MB solution (100 mg L^−1^). The adsorbent dosage gradient was 0.10–0.18 g. The suspensions were agitated at 120 rpm in a thermostatic shaker for 12 h. Following centrifugation (4000 rpm, 10 min), supernatant absorbance was measured at λ_max_ = 664 nm using UV–Vis spectroscopy.

### 2.5. Adsorption Kinetic and Isotherm Experiments

Adsorption kinetics and isotherms were measured using a 150 mL conical flask filled with 50 mL of MB solution (100 mg L^−1^). After adjusting the pH of the solution to 7, the system was pre-equilibrated for 10 min at 30 °C in a constant temperature shaker. The adsorbent (WS-1/WS-2), preheated to 30 °C, was added to the solution at a fixed dose of 1.4 in a single step to start the adsorption process immediately. Samples were taken at predetermined time points (0, 10, 30, 60, 120, 180 min), quickly filtered through a 0.22 μm membrane, and the concentration of residual MB (λ*_max_* = 664 nm) was determined immediately by UV–visible spectrophotometry.

## 3. Results and Discussion

### 3.1. Adsorbent Characterization

#### 3.1.1. Scanning Electron Microscope Images

**Figure 2:**
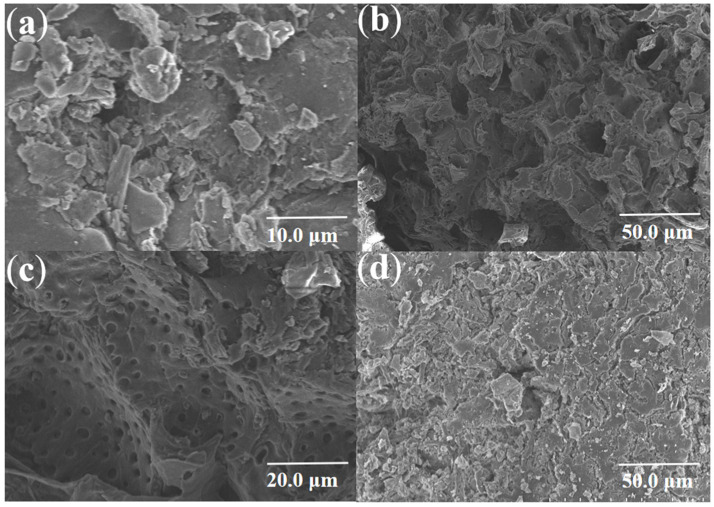
Scanning electron microscopy images of (**a**) WS, (**b**,**c**) WS-1 and (**d**) WS-2.

Figure 2a reveals a heterogeneous surface morphology with amorphous characteristics. Figure 2b,c demonstrate a well-distributed mesoporous architecture on WS-1 surfaces, indicating that LiP treatment effectively induced homogeneous pore formation through controlled delignification. In contrast, Figure 2d shows significant pore shrinkage and structural collapse in most enzymatically generated pores after subsequent hydrothermal carbonization.

#### 3.1.2. Fourier-Transform Infrared Spectroscopy Spectra

**Figure 3:**
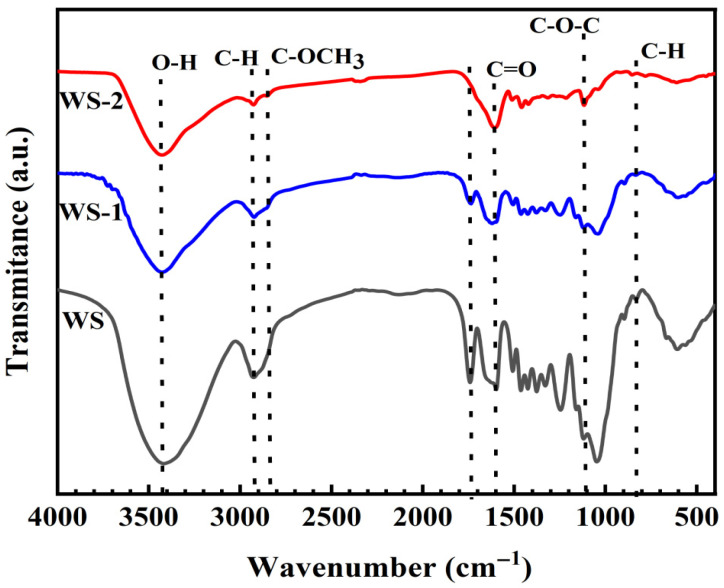
FT-IR spectra of WS, WS-1, and WS-2.

FT-IR analysis (Figure 3) revealed characteristic absorption bands at 3432 cm^−1^ (O–H stretching vibrations), 2942 cm^−1^ (C∓H stretching), and 1000–1500 cm^−1^ (C−O/O∓H deformations in alcoholic/phenolic hydroxyl, ether, and ester groups) [17]. Compared to pristine WS, WS-1 exhibits attenuated peak intensities at these characteristic regions, particularly the O∓H (3432 cm^−1^) and C∓H (2942 cm^−1^) stretching modes. This phenomenon can be attributed to partial lignin degradation via LiP treatment, which selectively cleaves aromatic polymer networks. The biomass components exhibited distinct architectures: hemicellulose comprises heterogeneous polysaccharide networks of diverse monosaccharides, cellulose consists of unbranched β-1,4-glycosidic bonded D-glucose chains, and lignin forms cross-linked macromolecules through C∓C bonds and ether linkages between phenylpropanoid subunits [18]. This structural hierarchy accounts for the comparatively lower thermal stability of hemicellulose [19]. Hydrothermal carbonization of WS-2 induces further O∓H/C∓H signal attenuation and disappearance at 1740 cm^−1^ (hemicellulose decomposition) [17]. Concurrently, the enhanced absorptions at 2850 cm^−1^, 1614 cm^−1^, and 1120 cm^−1^ suggest lignin stabilization and oxygen-rich functional group formation [20].

#### 3.1.3. Brunauer–Emmett–Teller Surface Area Analysis

**Figure 4:**
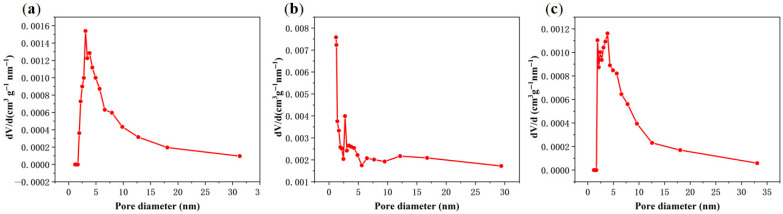
(**a**–**c**) Pore size distribution curves for WS, WS-1, and WS-2.

Specific surface area measurements (Table 1) showed a hierarchical evolution of porosity, where WS-2 > WS-1 > WS, a trend consistent with the morphological evolution observed by scanning electron microscopy. Pore size distribution profiles (Figure 4) show the following: (1) WSs with narrow micropore–mesopore ranges (peak < 5 nm), (2) WS-1 dominated by 5–15 nm mesopores, and (3) WS-2 exhibiting micropore concentration due to hydrothermal-induced macropore collapse [21].

#### 3.1.4. X-Ray Diffraction Patterns

**Figure 5:**
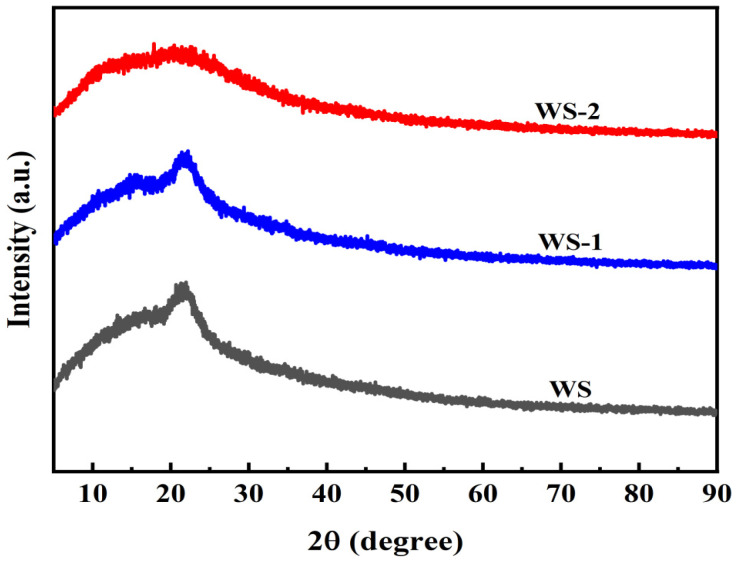
Comparative X-ray diffraction patterns of WS, WS-1, and WS-2.

Figure 5 presents the X-ray diffraction (XRD) patterns of WS, WS-1, and WS-2. It is revealed that all specimens exhibit characteristic cellulose diffraction signals within the 2θ range of 16–25°, corresponding to (002) and (040) crystallographic planes of cellulose *I_β_* [22]. More specifically, the WS displays a broad and indistinct diffraction peak in this region, indicative of a predominant amorphous structure arising from a disordered LCC matrix [23]. This observation aligns with native lignocellulosic biomass characteristics, where lignin’s amorphous nature suppresses cellulose crystallinity. As expected, WS-1 demonstrates marginally enhanced peak intensity, suggesting partial reduction in amorphous content through enzymatic modification. This phenomenon is attributed to selective delignification by LiP, which removes amorphous lignin components and partially exposes cellulose microfibrils [24]. Nevertheless, the broadened peaks confirm retention of the predominantly amorphous structure. In contrast, WS-2 exhibits significantly attenuated and flattened diffraction features, evidencing substantial cellulose crystallinity destruction and enhanced amorphization via hydrothermal carbonization. Hydrothermal conditions (elevated temperature and pressure) induce cellulose chain hydrolysis carbonization, coupled with hemicellulose degradation and lignin rearrangement [25]. These structural transformations correlate with oxygenated functional group generation and pore architecture development, synergistically enhancing adsorption capacity.

#### 3.1.5. Thermogravimetric Analysis Results

**Figure 6:**
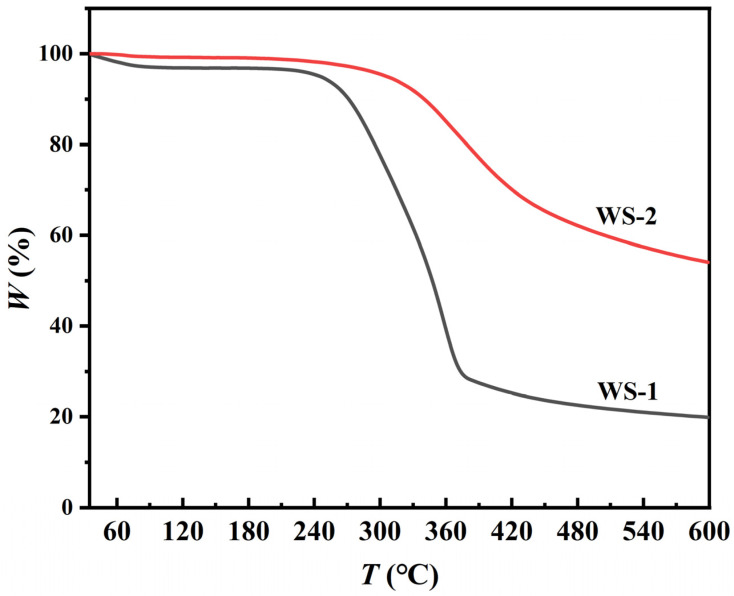
TGA analysis profiles of WS-1 and WS-2 under nitrogen atmosphere.

Figure 6 reveals distinct thermal degradation profiles of the specimens. WS-1 exhibits marginal weight loss (3.2%) at 0–120 °C, followed by rapid mass loss (68.9%) in the 260–360 °C range associated with cellulose decomposition. Above 480 °C, negligible mass loss (<8%) indicates carbonaceous residue stabilization, with total weight loss of 80.1%. In contrast, WS-2 demonstrates delayed decomposition onset at 330 °C with gradual 45.9% total mass loss, demonstrating superior thermal stability attributed to carbon matrix restructuring during hydrothermal treatment.

### 3.2. Adsorption Performances

#### 3.2.1. pH-Dependent Adsorption Behavior

**Figure 7:**
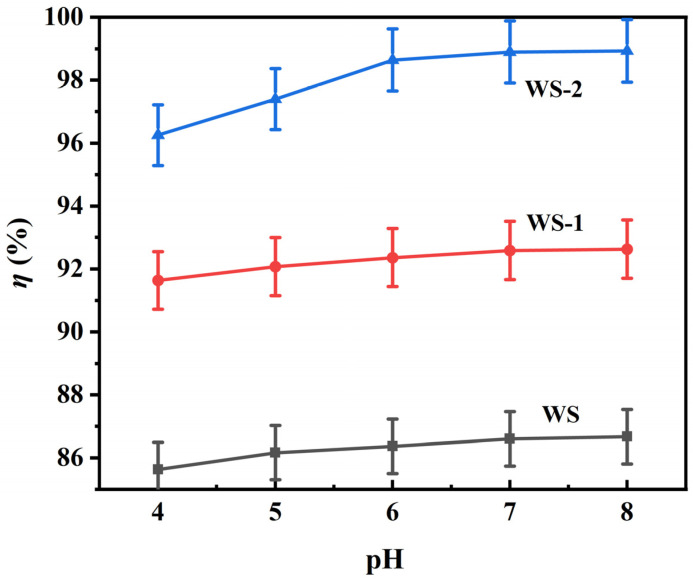
Effect of pH on MB adsorption. The amount of adsorbent used was 0.14 g, and the initial concentration of MB was 100 mg L^−1^.

Figure 7 demonstrates the pronounced pH-dependent adsorption behavior of WS-1 and WS-2 under the following fixed conditions: 0.14 g adsorbent dosage in 50 mL of 100 mg L^−1^ MB solution. This regulatory mechanism originates from surface charge modulation. Specifically, under acidic conditions (pH < 5), protonation induces positive surface charges and creates electrostatic repulsion with cationic MB^+^ species. Concurrently, H^+^ ions compete with MB^+^ for adsorption sites, resulting in suppressed uptake efficiency [26,27]. Progressive surface deprotonation occurs with pH elevation (6–10), increasing negative charge density. This charge reversal enhances electrostatic attraction with MB^+^ while improving accessibility to oxygen-containing functional groups (e.g., carboxyl, hydroxyl), thereby boosting both adsorption capacity and removal efficiency at a fixed initial MB concentration [28].

#### 3.2.2. Temperature-Dependent Adsorption Profiles

**Figure 8:**
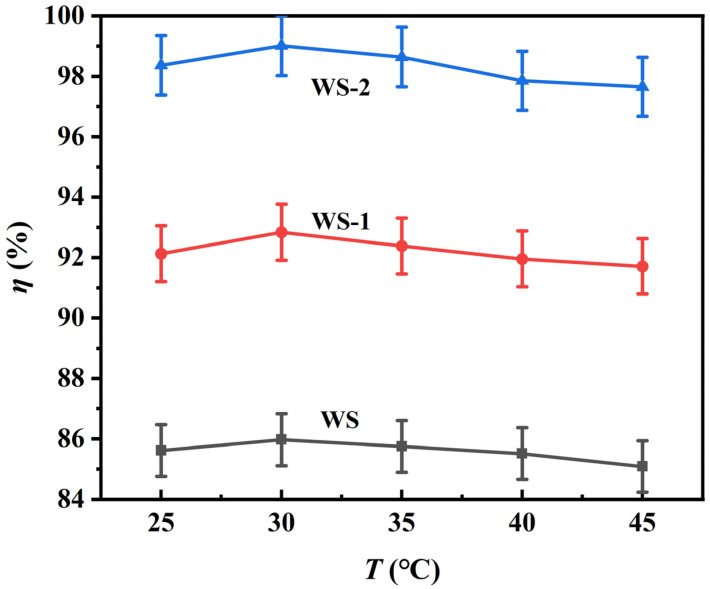
Temperature-dependent adsorption efficiency of the WS specimens. The amount of adsorbent used was 0.14 g, and the initial concentration of MB was 100 mg L^−1^.

Figure 8 demonstrates the temperature-dependent adsorption behavior of the specimens under fixed experimental conditions: 50 mL of 100 mg L^−1^ MB solution and 0.14 g adsorbent dosage, indicating that the best adsorption efficiency was achieved at 30 °C within the tested temperature range (25–45 °C). Below this threshold, elevated thermal energy enhances MB molecular diffusivity and collision frequency, thereby accelerating adsorption kinetics. When the temperature exceeds 30 °C, the adsorption efficiency declines sharply due to the exothermic nature of the process (Δ*H* = −28.5 kJ mol^−1^) [29], where increased thermal motion promotes desorption. Notably, WS-2 demonstrates more pronounced temperature sensitivity compared to WS-1, with a significant reduction in adsorption efficiency under thermal stress. This differential behavior stems from the adsorption mechanism of WS-2 dominated by carboxyl groups. The high temperatures induce two synergistic effects: enhanced molecular desorption through intensified thermal motion, and weakened electrostatic interaction between deprotonated carboxyl groups (-COO^−^) and the quaternary ammonium part of MB (N^+^(CH_3_)_2_), which jointly change the adsorption–desorption equilibrium [30]. Most importantly, the optimum temperature of 30 °C is suitable for industrial applications. Unlike energy-intensive modifications, such as the 180 °C WS-2 hydrothermal process, maintaining 30 °C requires minimal energy input while achieving >93% removal.

#### 3.2.3. Time-Resolved Adsorption Kinetics

**Figure 9:**
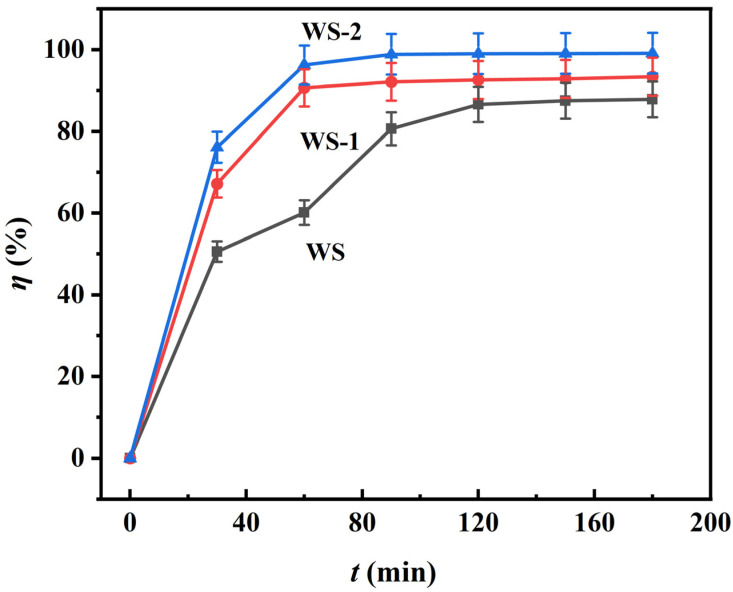
Adsorption kinetics as a function of contact time. The amount of adsorbent used was 0.14 g, and the initial concentration of MB was 100 mg L^−1^.

Time-dependent adsorption profiles of the specimens from kinetic studies are shown in Figure 9. All specimens exhibit rapid initial uptake followed by progressive rate attenuation, reaching equilibrium at 60 min (WS-1/WS-2) and 120 min (WS). This biphasic behavior stems from the initial abundance of surface-active sites facilitating rapid MB binding. Subsequent site occupancy and intermolecular repulsion between adsorbed free MB species progressively decelerate adsorption until site saturation [31]. Equilibrium removal efficiencies reach 93% (WS-1), 98% (WS-2), and 86% (WS), demonstrating significant performance enhancement through material modifications. Notably, WS-2 demonstrates exceptional adsorption efficiency (98% at equilibrium) through synergistic effects of carboxyl-functionalized electrostatic binding sites and optimized hierarchical porosity. In contrast, WS-1 achieves rapid equilibration (60 min) via LiP-induced macro-porous mass transfer pathways. This kinetic dichotomy elucidates the temporal regulation mechanism of adsorption through surface chemical modification.

#### 3.2.4. Adsorbent Dosage Optimization

**Figure 10:**
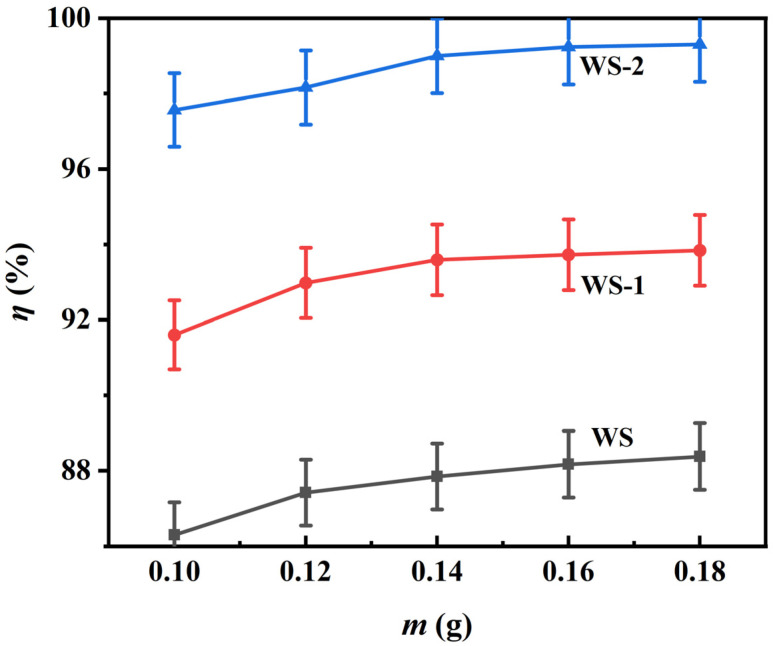
Dosage optimization study for methylene blue removal. The initial concentration of MB was 100 mg L^−1^.

Figure 10 shows the dose-dependent adsorption behavior for the removal of MB from 50 mL of 100 mg L^−1^ MB solution. Increasing the dosage of adsorbent expands the availability of active sites and thus improves the removal efficiency. However, beyond the critical threshold of 0.14 g, an increase in dosage provides only a weak improvement due to active site redundancy and reduced MB uptake. Saturation of such sites decreases the equilibrium adsorption density under conditions of fixed MB mass. From the point of view of economic optimization, 0.14 g is the optimal dosage for balanced system efficiency and resource utilization for a given MB concentration.

### 3.3. Adsorption Mechanisms

#### 3.3.1. Adsorption Kinetic Modeling

The kinetics of MB adsorption were analyzed using pseudo-first-order and pseudo-second-order models [32,33]. The adsorption capacity (*q_t_*) was calculated via Equation (1), followed by linear regression analysis with Equations (3) and (5) for pseudo-first-order and pseudo-second-order kinetics, respectively. This comparative modeling approach elucidates the adsorption mechanisms through regression coefficients (*R^2^*).

Adsorption capacity calculation formula:

(1) $$q_{t}=\frac{\left(c_{0}-c_{t}\right)}{m}V$$

Pseudo-first-order kinetic model formula [34]:

(2) $$q_{t}=q_{e}\left(1-e^{K_{1}t}\right)$$

(3) $$\ln\left(q_{e}-q_{t}\right)=\ln q_{e}-K_{1}t$$

Pseudo-second-order model formula [35]:

(4) $$q_{t}=\frac{K_{2}q_{e}^{2}t}{1+K_{2}q_{e}t}$$

(5) $$\frac{t}{q_{t}}=\frac{1}{K_{2}q_{e}^{2}}+\frac{t}{q_{e}}$$

where *c*_0_ represents the initial concentration, mg L^−1^. *c_t_* represents the concentration at the moment of *t*, mg L^−1^. *V* represents the volume of the solution, L. *m* represents the mass of the adsorbent, g. *t* is the adsorption time, min. *q_t_* represents the adsorbed amount at the moment of *t*, mg g^−1^. *q_e_* represents the saturated adsorbed amount at the time of equilibrium, mg g^−1^. *K*_1_ and *K*_2_ are pseudo-first-order and pseudo-second-order kinetic rate constants in min^−1^ and g mg^−1^ min^−1^, respectively.

**Figure 11:**
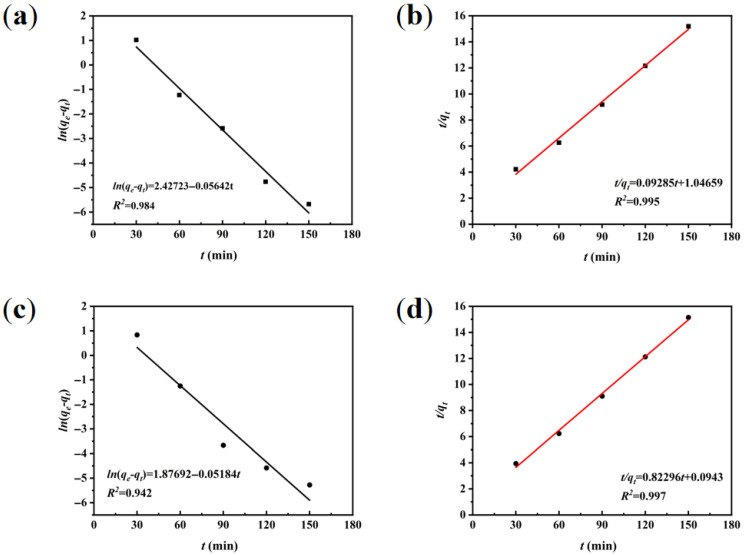
Linearized kinetic model fittings. (**a**,**b**) Pseudo-first-order and pseudo-second-order plots for WS-1, and (**c**,**d**) corresponding analyses for WS-2.

Figure 11 presents linearized kinetic plots for WS-1 (a,b) and WS-2 (c,d). The pseudo-second-order model demonstrates superior correlation coefficients (R^2^ > 0.98) compared to the pseudo-first-order model, indicating chemisorption-dominated mechanisms for both adsorbents. Therefore, the WS modifications successfully incorporated oxygen-rich functionalities (hydroxyl, carbonyl groups) that enhance MB binding through hydrogen bonding and electrostatic interactions with cationic dye moieties [36]. WS-1 demonstrates optimized pore architecture with enhanced micro-mesopore distribution, improving molecular diffusion pathways and active site accessibility compared to unmodified WS [37]. WS-2 exhibits further structural refinement with increased micropore dominance and specific surface area development, creating superior adsorption sites that account for its enhanced performance over WS-1.

#### 3.3.2. Adsorption Isotherm Modeling

At adsorption equilibrium, experimental data were fitted to Langmuir and Freundlich isotherm models. The Langmuir model assumes homogeneous adsorption surfaces with no intermolecular interactions, describing monolayer coverage through finite active sites [38]. In contrast, the Freundlich model characterizes multilayer adsorption on heterogeneous surfaces, demonstrating broader applicability across concentration gradients. The mathematical expressions are as follows:

Langmuir equation [39]:

(6) $$q_{e}=\frac{q_{m}K_{L}c_{e}}{1+K_{L}c_{e}}$$

(7) $$\frac{c_{e}}{q_{e}}=\frac{1}{q_{m}K_{L}}+\frac{c_{e}}{q_{m}}$$

Freundlich equation [40]:

(8) qe=KFce1n

(9) $$\ln q_{e}=\ln K_{F}+\frac{1}{n}\ln c_{e}$$

where *q_e_* is the equilibrium adsorption amount, mg g^−1^. *c_e_* is the concentration of the solution when the reaction reaches equilibrium, mg L^−1^. *q_m_* is the maximum adsorption amount, mg g^−1^. *K_L_* and *K_F_* are Langmuir and Freundlich’s adsorption affinity constants L mg^−1^, respectively, and *n* denotes the adsorption strength factor.

**Figure 12:**
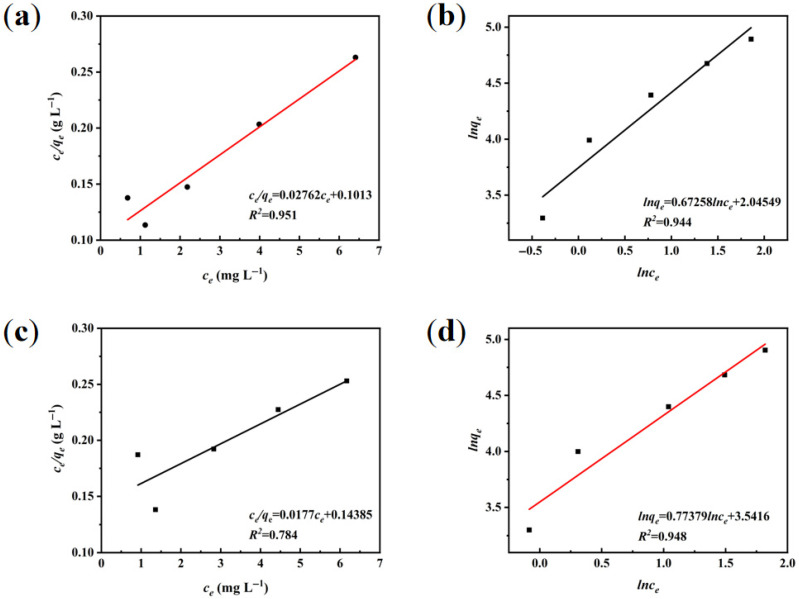
Isotherm model regression analysis. (**a**,**b**) Langmuir vs. Freundlich fittings for WS-1, and (**c**,**d**) comparative models for WS-2.

Figure 12a,b shows linearized Langmuir (*R*^2^ = 0.951) and Freundlich (*R*^2^ = 0.944) isotherm fittings for WS-1. The higher Langmuir correlation coefficient suggests monolayer adsorption dominance. Enzymatic oxidation generates surface −OH and C=O groups that facilitate hydrogen bonding and π-π interactions. While macroporous architecture enhances mass transfer, limited surface area concentrates adsorption capacity at accessible active sites. For WS-2 (Figure 12c,d), the Freundlich model demonstrates superior correlation (*R^2^* = 0.948) compared to the Langmuir model (*R*^2^ = 0.784), indicating heterogeneous multilayer adsorption characteristics. Hydrothermal modification with citric acid introduces −COOH groups onto WS-2 [41]. These groups enhance the surface electronegativity and give the cationic groups of MB a strong electrostatic attraction. Therefore, this is a process driven by chemisorption [42]. Although partial macropore collapse occurs, emerging micro-mesopores enhance surface area potential. Coupled with non-uniform functional group distribution, this creates heterogeneous adsorption interfaces. The simulated *q_e_* values (34.5 mg g^−1^ for WS-1 and 36.1 mg g^−1^ for WS-2, respectively) showed excellent agreement with the experimental measurements (33.57 mg g^−1^ and 35.7 mg g^−1^, respectively) with a deviation of <3%. This confirms the plausibility of pseudo-secondary kinetics in characterizing MB adsorption at different temperatures.

### 3.4. Analysis of Regenerative Effects

**Figure 13:**
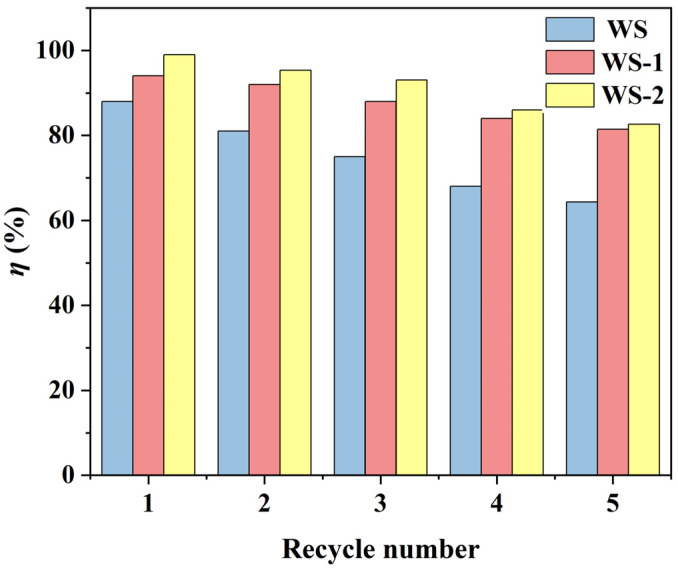
Desorption and regeneration effects of WS and WS-1, WS-2.

Figure 13 shows the variation in MB removal efficiency for WS, WS-1, and WS-2 over successive regeneration cycles. The MB removal rate showed a decreasing trend as the number of cycles increased. After five cycles, the removal of MB decreased to 81.45% and 82.67% for WS-1 and WS-2, respectively. Although the MB removal decreased, it still remained above 80%, which indicated the good reusability of the modified adsorbents. The MB removal of WS decreased to 64.32%, which indicated that the reusability of unmodified WS was relatively poor. In practical applications, WS-1 and WS-2 can effectively remove MB and can be reused several times after regeneration, which possess good environmental and economic benefits.

The adsorption capacities of several different types of biochar for MB are presented in Table 2. It illustrates the adsorption capacity of biochars prepared from different biomass feedstocks, at various pyrolysis temperatures, and under different modification conditions. In this study, WS exhibits adsorption potential. After enzymatic modification (WS-1) and hydrothermal carbonization with citric acid (WS-2), the adsorption performance was further improved, among which WS-2 achieved 99.01% removal (measured adsorption 35.7 mg g^−1^) under optimized conditions, which was 16% higher than that of WS. Although WS-2 was slightly more efficient in removing methyl bromide (99.01% vs. 93.67%), its hydrothermal carbonization process is much more energy-intensive compared to the enzymatic modification of WS (WS-1).

Although WS-2 achieved marginally higher MB removal efficiency (99.01% vs. 93.67%), its hydrothermal carbonization at 180 °C requires significantly greater energy input than enzymatic modification of WS-1 at 40 °C, primarily due to the elevated temperature and pressure conditions. For large-scale wastewater treatment, the inherently lower energy demand of enzymatic processing may offset the 5.34% capacity gap (35.7 vs. 33.6 mg g^−1^), aligning with industrial priorities for sustainable operations that balance efficiency and resource consumption.

## 4. Conclusions

Highly efficient adsorption of MB was achieved using enzyme-modified walnut shells (WS-1) and the hydrothermally carbonized product derived from these walnut shells (WS-2). Under the conditions of an adsorbent dosage of 0.14 g, 50 mL of 100 mg L^−1^ MB solution, pH 7, 30 °C and an adsorption time of 120 min, the removal of MB by WS-1 and WS-2 reached 93.67% and 99.01%, respectively. The adsorption behavior of WS-1 is consistent with the pseudo-second-order kinetic model and Langmuir isotherm model, suggesting that the adsorption process is a synergistic interaction between weak chemisorption and physisorption on the surface. However, WS-2 conforms to the pseudo-second-order kinetic model and the Freundlich isotherm model, exhibiting multilayer chemisorption, which was attributed to the hydrothermal treatment increasing the specific surface area and surface functional groups (e.g., −COOH, −OH groups, etc.) of WS-2. The excellent performance of the environmentally friendly WS-derived materials in MB adsorption demonstrates its great potential for application in dye wastewater treatment, and provides a new idea for the valorization of low-value agricultural wastes.

## Figures and Tables

**Table 1:** Textural properties from BET analysis.

Adsorbents	BET Surface Area
WS	192 m^2^ g^−1^
WS-1	462 m^2^ g^−1^
WS-2	630 m^2^ g^−1^

**Table 2:** Comparison of MB adsorption capacity by different types of biochar.

Type of Biochar	Pyrolysis Temperature (°C)	Modifying Agent	Adsorption Capacity (mg g^−1^)	Reference
Pine wood	525	Physical pulverization	25	[43]
Pig manure	400	Physical pulverization	7.9	[43]
Cardboard	500	Physical pulverization	8.9	[43]
Rice straw	500	Ball milling	50.27	[44]
Cotton residue	550	NaOH	23.82	[45]
Tea residue	700	NaOH	105.44	[46]
WS	25	None	31.4	Present study
WS-1	40	Lignin peroxidase	33.57	Present study
WS-2	180	Citric acid	35.7	Present study

## Data Availability

The original contributions presented in this study are included in the article. Further inquiries can be directed to the corresponding authors.
